# Predictors of burnout among HIV nurses in the Western Cape

**DOI:** 10.4102/curationis.v40i1.1695

**Published:** 2017-06-28

**Authors:** Rizwana Roomaney, Jeanette Steenkamp, Ashraf Kagee

**Affiliations:** 1 Department of Psychology, Stellenbosch University, South Africa

## Abstract

**Background:**

Burnout has been implicated as one of the reasons for key healthcare personnel, such as nurses, leaving their profession, resulting in insufficient staff to attend to patients.

**Objective:**

We investigated the predictors of three dimensions of burnout, namely emotional exhaustion, depersonalisation and personal accomplishment, among nurses in South Africa attending to patients living with HIV.

**Method:**

Participants were recruited at a large tertiary hospital in the Western Cape region, with the help of the assistant director of nursing at the hospital. They completed the Maslach Burnout Inventory, the Quantitative Workload Inventory, the Interpersonal Conflict at Work Scale, the Organisational Constraints Scale, the Death and Dying subscale of the Nursing Stress Scale, and the HIV and AIDS Stigma Instrument – Nurse.

**Results:**

We found elevated levels of burnout among the sample. Workload, job status and interpersonal conflict at work significantly explained more than one-third of the variance in emotional exhaustion (*R*² = 0.39, F(7, 102) = 9.28, *p* = 0.001). Interpersonal conflict, workload, organisational constraints and HIV stigma significantly explained depersonalisation (*R*² = 0.33, F(7, 102) = 7.22, *p* = 0.001). Job status and organisational constraints significantly predicted personal accomplishment (*R*² = 0.18, F(7, 102) = 3.12, *p* = 0.001).

**Conclusion:**

Factors such as workload, job status and interpersonal conflict in the work context, organisational constraints and stigma associated with HIV were found to be predictors of burnout in the sample of nurses. Our recommendations include developing and testing interventions aimed at reducing burnout among nurses, including reducing workload and creating conditions for less interpersonal conflict at work.

## Introduction

### Problem statement

Burnout has been implicated as one of the reasons for key healthcare personnel such as nurses leaving their profession, resulting in insufficient staff to attend to patients (Aiken et al. 2002). Nurses in the South African public healthcare system face a range of challenges that create conditions under which burnout occurs. These conditions include being overworked, having to deal with a large number of patients in a short period of time, poor hospital facilities and infrastructure and non-competitive remuneration (Bester & Engelbrecht 2009; Rispel & Bruce 2014). Among HIV nurses, burnout may be exacerbated because of the challenging nature of this patient population, for example multiple hospitalisations, poor treatment adherence and the high likelihood of mortality. Identifying predictors of burnout may be helpful in monitoring nurses in order to prevent burnout. Therefore, this study sought to identify the psychosocial correlates of burnout among HIV nurses in the Western Cape province of South Africa.

### Literature review

Burnout refers to ‘a syndrome of emotional exhaustion, depersonalization and reduced personal accomplishment that can occur among individuals who work with people in some capacity’ (Schaufeli, Leiter & Maslach 2009:206). There are three dimensions associated with burnout, namely emotional exhaustion, depersonalisation and reduced personal accomplishment. Emotional exhaustion involves the feeling of being overextended and depleted of emotional resources (Maslach & Goldberg 1998). Depersonalisation refers to responding negatively or in an excessively detached way towards others and may include a loss of idealism (Maslach & Goldberg 1998). Reduced personal accomplishment refers to a reduction in feelings of competence and productivity in the work context (Maslach & Goldberg 1998). Burnout results from disharmony between an individual and his or her work environment in terms of the various work life areas, namely, workload, control, reward, community, fairness and values (Leiter & Maslach 1999). Burnout has been cited as one of the key reasons for nurses’ intention to leave the profession (Dimattio, Roe-prior & Carpenter 2010). Among South African nurses, such intentions were related to elevated levels of burnout, specifically emotional exhaustion and depersonalisation (Pienaar & Bester 2011).

### Factors associated with burnout

A number of factors have been associated with burnout. These factors are workload, interpersonal conflict, organisational constraints, dealing with death and dying, secondary HIV stigma and demographic characteristics. The research for each of these stressors is discussed below.

#### Workload

In the nursing profession, in general, it has been noted that workload is a major workplace stressor (Glazer & Gyurak 2008). This is especially true in South Africa, where HIV and AIDS places a greater burden on carers and, thus, an increased workload among hospital nurses (Coetzee et al. 2013; De Wet & Du Plooy 2012).

With over 5 million persons living with HIV, the sheer number seeking treatment creates overcrowded conditions in clinics (Coetzee, Kagee & Vermeulen 2011), resulting in an inordinate workload for nurses. Specific additional duties to caring for patients receiving antiretroviral therapy (ART), such as providing adherence counselling, assisting patients in obtaining social grants and advising on ways to prevent medication side effects, add substantially to nurses’ existing workload (De Wet & Du Plooy 2012).

#### Interpersonal conflict

It has been reported that in some contexts, nurses come into conflict with other players in the clinic or hospital, such as their managers, other nurses, doctors, patients and patients’ relatives (Guidroz, Wang & Perez 2012). Numerous studies around the world have shown robust associations between interpersonal conflict and burnout in various contexts (García-Izquierdo & Ríos-Rísquez 2012; Guidroz et al. 2012; Kitaokai & Masuda 2012).

#### Organisational constraints

A lack of adequate resources including lack of supplies, lack of equipment, broken equipment and unavailable resources has been shown to be among the top workplace stressors in several countries (Glazer & Gyurak 2008). In South Africa, poor workplace infrastructure and the unavailability of medical supplies were key reasons health workers either left the public health sector or emigrated from South Africa (Pendleton, Crush & Lefko-Everett 2007). Organisational constraints, workload and interpersonal conflict at work have been robustly associated with emotional exhaustion, a key aspect of burnout (Engelbrecht et al. 2008).

#### Death and dying

Engaging with the emotional issues around death or dying is a strong predictor of burnout (Garrosa et al. 2008). Nearly 90% of South African nurses caring for persons living with HIV empathised strongly with their patients and found it difficult to see patients experience suffering and ultimately death (Davhana-Maselesele & Igumbor 2008).

#### Secondary HIV and AIDS stigma

HIV-related stigma has been defined as ‘a “process of devaluation” of people either living with or associated with HIV and AIDS’ (UNAIDS 2007:9). Secondary HIV and AIDS stigma experienced as a result of being associated with people living with HIV (PLWH) has been identified as an important occupational stress factor among nurses (Haber, Roby & High-George 2011). HIV caregivers, including nurses, have been ostracised by community members and even by friends and family (Van Dyk 2007). For example, among nurses in five African countries, including South Africa, perceived HIV and AIDS stigma was shown to predict job dissatisfaction (Chirwa et al. 2009).

#### Demographic characteristics

Demographic factors such as age and job status have been found to influence the intensity of burnout among nurses (Garrosa et al. 2008). For example, among a sample of Spanish nurses, age and job status accounted for 16%, 14% and 12% of the variance in emotional exhaustion, depersonalisation and personal accomplishment, respectively (Garrosa et al. 2008). Younger nurses in the study experienced greater levels of burnout than older nurses.

In South Africa, high levels of emotional exhaustion were found among nurses who worked both in public and private hospitals (Coetzee et al. 2013), with 45.8% scoring in the elevated range (greater than 27) on the Maslach Burnout Inventory (MBI). Further, 53.8% of the participants in Coetzee’s study who worked in public hospitals scored in the high range for emotional exhaustion compared with 40.6% who worked in private hospitals. Similarly, in a study of 818 South African nurses, 34.6% and 20.4% of participants scored in the elevated range on emotional exhaustion and depersonalisation, respectively (Van der Colff & Rothmann 2012). High levels of burnout were also found in a study of over 500 nurses from the Free State province in South Africa, with reported mean scores of 31.34 for emotional exhaustion and 17.80 for depersonalisation (Engelbrecht et al. 2008). Additionally, 68.7% and 85.1% of participants reported elevated levels of emotional exhaustion and depersonalisation, respectively. Among 935 critical care nurses in private and public South African hospitals, the mean score of 27.04 on the MBI fell in the elevated range, with 59.4% scoring in the moderate range for emotional exhaustion, and 16.4% scoring in the high range (Klopper et al. 2012). Further, 68.5% and 3% of the sample reported moderate and high levels of depersonalisation, respectively (Bruce & Sangweni 2012). The research indicates that nurses experience a high rate of burnout. Furthermore, a number of factors have been associated with burnout in previous research: that is, age, job status, workload, interpersonal conflict at work, organisational constraints, death and dying-related stress, and HIV and AIDS stigma by association. The aim of this study was to test the ability of these factors to predict burnout.

#### Research objective

In the context of the challenging terrain of HIV treatment, we investigated the predictors of three dimensions of burnout, namely emotional exhaustion, depersonalisation and personal accomplishment among a sample of South African nurses caring for patients living with HIV. We tested the predictors (i.e. age, job status, workload, interpersonal conflict at work, organisational constraints, death and dying-related stress and HIV and AIDS stigma by association) on each of these dimensions of burnout. The objectives of the study were as follows:

- to determine the extent to which age, job status, workload, interpersonal conflict at work, organisational constraints, death and dying-related stress and HIV and AIDS stigma by association were predictors of emotional exhaustion
- to determine the extent to which age, job status, workload, interpersonal conflict at work, organisational constraints, death and dying-related stress and HIV and AIDS stigma by association were predictors of depersonalisation
- to determine the extent to which age, job status, workload, interpersonal conflict at work, organisational constraints, death and dying-related stress and HIV and AIDS stigma by association were predictors of personal accomplishment.

## Research method and design

### Design

The study consisted of a cross-sectional design and the data were collected from nurses using psychometric measures. Participants were nurses in various departments at a large tertiary hospital outside Cape Town. We computed the sample size using the G*Power statistical programme (Faul et al. 2007). Thus, a sample of 109 participants was required to support the multiple regression analysis with seven predictors, assuming a medium effect size of 0.20, power of 0.93 and 95% significance level.

### Materials

The instruments were compiled into data collection booklets that contained questions relating to demographic information and contained the five psychometric instruments. The measure was self-administered.

#### Demographic information

Age, gender, race, marital status, living situation, education level, job status, time in the nursing profession, work details, income level, place of birth and languages spoken were obtained using a demographic questionnaire.

#### Burnout

The 22-item MBI was used to assess three components of burnout, namely emotional exhaustion, depersonalisation and personal accomplishment (Maslach, Jackson & Leiter 1996). Respondents were asked to indicate the frequency of their experiences, specifically their feelings and attitudes, on a 7-point Likert scale with response options ranging from 0 (never) to 6 (every day). High scores on the emotional exhaustion and depersonalisation dimensions correspond to higher levels of burnout and high scores on the personal accomplishment dimension refer to lower levels of burnout. Maslach et al. (1996) have reported high internal consistency for the three subscales with the alpha reliabilities ranging from 0.71 to 0.90. The MBI has also been used in other South African studies to examine burnout among nurses, with high alpha coefficients for the various subscales (Coetzee et al. 2013; Engelbrecht et al. 2008). For this study, the internal consistency of the MBI as measured by Cronbach’s alpha was 0.85. Cronbach’s alphas for emotional exhaustion, depersonalisation and personal accomplishment subscales were 0.85, 0.60 and 0.67 respectively.

#### Workload

The Quantitative Workload Inventory (QWI) was used to measure the perceived amount of work (Spector & Jex 1998). The QWI has five items which ask about the amount and pace of work. High perceived workload is represented by high scores. High alpha coefficients have been consistently reported for this scale. For this study, the internal consistency of the QWI as measured by Cronbach’s alpha was 0.80.

#### Interpersonal conflict

The four-item Interpersonal Conflict at Work Scale (ICAWS) measured participants’ perceived level of interpersonal conflict at work, which ranges from insignificant personal differences to physical assaults and which can be either overtly or covertly expressed (Spector & Jex 1998). The ICAWS has demonstrated high internal consistency in several studies (Kath et al. 2013). Cronbach’s alpha for the ICAWS in this study was 0.78.

#### Organisational constraints

The 11-item Organisational Constraints Scale (OCS) measured the extent to which participants experienced organisational constraints at work. Examples include situations that interfere with job performance such as ‘Inadequate training’, ‘Poor equipment or supplies’, and ‘Inadequate help from others’ (Spector & Jex 1998). The instrument asks respondents to indicate how often the item makes job performance difficult or impossible (Spector & Jex 1998). The range of response options is from 1 (less than once per month or never) to 5 (several times per day). High scores correspond to more organisational constraints. High alpha coefficients have been reported in various studies (Kath et al. 2013). The Cronbach’s alpha for this study was 0.89.

#### Stress

Death and dying-related stress were measured by the Death and Dying subscale of the Nursing Stress Scale (NSS). The NSS has demonstrated high internal consistency in various studies (Rosnawati et al. 2010). For this study, the internal consistency of the NSS as measured by Cronbach’s alpha was 0.86.

#### HIV and AIDS stigma

The 19-item HIV and AIDS Stigma Instrument – Nurse (HASI-N) was used to measure nurses’ perpetration and experiences related to HIV-related stigma (Uys et al. 2009). We used the nine items of the Nurses Being Stigmatised factor to measure HIV and AIDS stigma by association as experienced by the nurses. The scale has shown high internal consistency (Kohi et al. 2010). For this study, the internal consistency of the HASI-N as measured by Cronbach’s alpha was 0.91.

### Procedure

The flyers were distributed to the nurses by their supervisors who asked for the contact details of those who wished to participate in the study. These contact details were then given to the researchers. A research assistant then approached each nurse who then completed the questionnaire. We continued to recruit participants until the required sample size was obtained. Participants completed the paper and pencil questionnaires either outside of working hours or during breaks at work. Participants were given a voucher as a token of gratitude for their participation. Following a pilot study among six respondents to ensure comprehensibility of the items in both English and Afrikaans, a total of 123 participants was recruited into the study, and 110 provided complete questionnaires.

### Data analysis

We computed the Pearson’s product moment correlation coefficients between the three dimensions of burnout (emotional exhaustion, depersonalisation and personal accomplishment) and job status, age, death and dying-related stress, workload, organisational constraints, interpersonal conflict at work and HIV and AIDS stigma by association.

Three separate multiple regression models were computed. We used a hierarchical method of entry and entered predictor variables in terms of theoretical importance. In the first model, we regressed emotional exhaustion onto the predictor variables in the following order: age, job status, workload, interpersonal conflict at work, organisational constraints, death and dying-related stress and HIV and AIDS stigma. In the second model, we regressed depersonalisation onto the predictor variables. In the third model, we regressed personal accomplishment onto the predictor variables. A new predictor variable was added at each step, with the final model containing all the seven predictors.

### Ethical consideration

Approval for the study was obtained from the Stellenbosch University Health Research Ethics Committee and permission to collect the data was obtained from the Western Cape Department of Health. All participants completed informed consent forms. Nurses were informed of their right to terminate participation and were assured that the data derived from the study will be treated confidentially. Participants were provided with the details of free counselling services as a referral source, if any participant felt distressed following participation in the study. Completed questionnaires and consent forms were stored in a locked drawer for five years following completion of the study.

## Results

A total of 110 participants submitted completed questionnaires, the majority of whom were female (*n* = 106; 96.4%), ‘coloured’ (*n* = 79; 71.8%) and Afrikaans-speaking (*n* = 82; 74.5%). The average age of the sample was 42.81 years (standard deviation = 8.82 years). The majority of the sample (61.8%) reported being older than 40 years. Almost half of the participants (45.4%) worked as nurses for more than 20 years (median age = 44.00 years). The sample consisted of 5 (4.5%) nurses completing community service, 40 (36.4%) enrolled nursing assistants, 30 (27.3%) enrolled nurses and 35 (31.8%) professional nurses. Most of the sample (64.5%) worked most frequently on the day shift. Participants reported working in a number of wards such as the adult medical and surgical ward (30%), multiple wards (20%), casualty ward (14.5%), paediatric ward (10%), maternity ward (10%), outpatient ward (6.4%), theatre (4.5%) and high-care ward (1.8%) and in offices (2.7%).

### Predictors of burnout

The results of the regression analysis are presented in Tables 1–6. Tables 1–3 contain the model summaries for the dimensions of burnout. Tables 4–6 contain the beta coefficients for variables in final models.

**TABLE 1:** Multiple regression analyses for variables predicting emotional exhaustion.

Model	*R*	*R*^2^	Δ*R*^2^	SE	*F*	*df1*	*df2*	*p*
1^a^	0.49	0.24**	0.24**	10.50	34.45	1	108	0.00
2^b^	0.56	0.31**	0.07**	10.07	23.87	2	107	0.00
3^c^	0.58	0.33**	0.03*	9.93	17.71	3	106	0.00
4^d^	0.58	0.34**	0.00	9.96	13.30	4	105	0.00
5^e^	0.59	0.35**	0.01	9.93	11.02	5	104	0.00
6^f^	0.59	0.35**	0.00	9.98	9.10	6	103	0.00
7^g^	0.62	0.39**	0.04**	9.70	9.28	7	102	0.00

*Source:* Authors’ own work

*N* = 110.

*R*, correlation coefficient; *R*^2^, coefficient of determination; Δ*R*², R squared change; SE, standard error of the estimate; *F*, F-ratio; *Df1*, degrees of freedom; *Df2*, degrees of freedom; *P*, significance.

a , Predictors: (Constant), Workload.

b , Predictors: (Constant), Workload, interpersonal conflict at work.

c , Predictors: (Constant), Workload, interpersonal conflict at work, organisational constraints.

d , Predictors: (Constant), Workload, interpersonal conflict at work, organisational constraints, age.

e , Predictors: (Constant), Workload, interpersonal conflict at work, organisational constraints, age, death and dying-related stress.

f , Predictors: (Constant), Workload, interpersonal conflict at work, organisational constraints, age, death and dying-related stress, HIV and AIDS stigma by association.

g , Predictors: (Constant), Workload, interpersonal conflict at work, organisational constraints, age, death and dying-related stress, HIV and AIDS stigma by association, job status.

* , *p* < 0.05;

** , *p* < 0.01

**TABLE 2:** Multiple regression analyses for variables predicting depersonalisation.

Model	*R*	*R*^2^	Δ*R*^2^	SE	*F*	*df1*	*df2*	*p*
1^a^	0.26	0.07**	0.07**	5.34	7.86	1	108	0.01
2^b^	0.46	0.21**	0.14**	4.94	14.19	2	107	0.00
3^c^	0.50	0.25**	0.04*	4.83	11.79	3	106	0.00
4^d^	0.51	0.26**	0.01	4.83	9.06	4	105	0.00
5^e^	0.51	0.26**	0.01	4.83	7.46	5	104	0.00
6^f^	0.54	0.30**	0.03*	4.75	7.19	6	103	0.00
7^g^	0.58	0.33**	0.04*	4.65	7.22	7	102	0.00

*Source:* Authors’ own work

*N* = 110.

*R*, correlation coefficient; *R*^2^, coefficient of determination; Δ*R*², *R* squared change; SE, standard error of the estimate; *F*, F-ratio; *Df1*, degrees of freedom; *Df2*, degrees of freedom; *P*, significance.

a , Predictors: (Constant), Workload.

b , Predictors: (Constant), Workload, interpersonal conflict at work.

c , Predictors: (Constant), Workload, interpersonal conflict at work, organisational constraints.

d , Predictors: (Constant), Workload, interpersonal conflict at work, organisational constraints, age.

e , Predictors: (Constant), Workload, interpersonal conflict at work, organisational constraints, age, death and dying-related stress.

f , Predictors: (Constant), Workload, interpersonal conflict at work, organisational constraints, age, death and dying-related stress, HIV and AIDS stigma by association.

g , Predictors: (Constant), Workload, interpersonal conflict at work, organisational constraints, age, death and dying-related stress, HIV and AIDS stigma by association, job status.

* , *p* < 0.05;

** , *p* < 0.01

**TABLE 3:** Multiple regression analyses for variables predicting personal accomplishment.

Model	*R*	*R*^2^	Δ*R*^2^	SE	*F*	*df1*	*df2*	*p*
1^a^	0.13	0.02	0.02	7.59	1.73	1	108	0.19
2^b^	0.16	0.02	0.01	7.59	1.34	2	107	0.27
3^c^	0.25	0.06	0.04*	7.49	2.27	3	106	0.09
4^d^	0.26	0.07	0.01	7.50	1.88	4	105	0.12
5^e^	0.29	0.08	0.02	7.47	1.85	5	104	0.11
6^f^	0.30	0.09	0.01	7.47	1.71	6	103	0.13
7^g^	0.42	0.18**	0.09**	7.15	3.12	7	102	0.01

*Source:* Authors’ own work

*N* = 110.

*R*, correlation coefficient; *R*^2^, coefficient of determination; Δ*R*², R squared change; SE, standard error of the estimate; *F*, F-ratio; *Df1*, degrees of freedom; *Df2*, degrees of freedom; *P*, significance.

a , Predictors: (Constant), Workload.

b , Predictors: (Constant), Workload, interpersonal conflict at work.

c , Predictors: (Constant), Workload, interpersonal conflict at work, organisational constraints.

d , Predictors: (Constant), Workload, interpersonal conflict at work, organisational constraints, age.

e , Predictors: (Constant), Workload, interpersonal conflict at work, organisational constraints, age, death and dying-related stress.

f , Predictors: (Constant), Workload, interpersonal conflict at work, organisational constraints, age, death and dying-related stress, HIV and AIDS stigma by association.

g , Predictors: (Constant), Workload, interpersonal conflict at work, organisational constraints, age, death and dying-related stress, HIV and AIDS stigma by association, job status.

* , *p* < 0.05;

** , *p* < 0.01

**TABLE 4:** Predictors of emotional exhaustion.

Variable	Model 1	Model 2	Model 3	Model 4	Model 5	Model 6	Model 7				*P*
	
	*β*	*β*	*β*	*β*	*β*	*β*	*β*	95% CI	SE *β*	*β*	
Constant	-	-	-	-	-	-	−14.94	(−28.15, −1.72)	6.66	-	0.027
Workload	0.49**	0.40**	0.35**	0.36**	0.36**	0.36**	0.77	(0.32, 1.21)	0.23	0.30**	0.001
Interpersonal conflict at work	-	0.27**	0.21*	0.21*	0.21*	0.21*	0.76	(0.10, 1.41)	0.33	0.20*	0.024
Organisational constraints	-	-	0.19*	0.18	0.16	0.16	0.21	(-0.02, 0.43)	0.11	0.17	0.072
Age	-	-	-	0.05	0.04	0.04	0.03	(−0.19, 0.24)	0.11	0.02	0.799
Death and dying-related stress	-	-	-	-	0.10	0.10	0.20	(−0.22, 0.62)	0.21	0.08	0.356
HIV and AIDS stigma by association	-	-	-	-	-	0.01	0.19	(−0.19, 0.56)	0.19	0.09	0.333
Job status	-	-	-	-	-	-	2.15	(0.55, 3.74)	0.81	0.23**	0.009

*Source:* Authors’ own work

*N* = 110.

*β*, beta; SE *β*, standard error of beta; CI, confidence interval; *P*, significance.

* , *p* < 0.05;

** , *p* < 0.01

**TABLE 5:** Predictors of depersonalisation.

Variable	Model 1	Model 2	Model 3	Model 4	Model 5	Model 6	Model 7				*P*
	
	*β*	*β*	*β*	*β*	*β*	*β*	*β*	95% CI	SE β	*β*	
Constant	-	-	-	-	-	-	−2.33	(−8.67, 4.01)	3.20	-	0.467
Workload	0.26**	0.13	0.07	0.06	0.07	0.07	0.02	(−0.20, 0.24)	0.11	0.02	0.853
Interpersonal conflict at work	-	0.40**	0.32**	0.31**	0.31**	0.28**	0.46	(0.15, 0.78)	0.16	0.27**	0.004
Organisational constraints	-	-	0.23*	0.24*	0.21*	0.20	0.12	(0.01, 0.22)	0.05	0.21*	0.034
Age	-	-	-	−0.08	−0.09	−0.09	−0.07	(−0.17, 0.04)	0.05	−0.11	0.203
Death and dying-related stress	-	-	-	-	0.09	0.04	0.02	(−0.19, 0.22)	0.10	0.01	0.880
HIV and AIDS stigma by association	-	-	-	-	-	0.19*	0.26	(0.08, 0.44)	0.09	0.26**	0.006
Job status	-	-	-	-	-	-	0.91	(0.14, 1.68)	0.39	0.21*	0.021

*Source:* Authors’ own work

Note: *N* = 110.

*β*, beta; SE *β*, standard error of beta; CI, confidence interval; *P*, significance.

* *p* < 0.05;

** *p* < 0.01

**TABLE 6:** Predictors of personal accomplishment.

Variable	Model 1	Model 2	Model 3	Model 4	Model 5	Model 6	Model 7				*P*
	
	*β*	*β*	*β*	*β*	*β*	*β*	*β*	95% CI	SE *β*	*β*	
Constant	-	-	-	-	-	-	42.21	(32.47, 51.94)	4.91	-	0.000
Workload	−0.13	−0.09	−0.04	−0.03	−0.04	−0.04	0.08	(−0.25, 0.41)	0.17	0.05	
Interpersonal conflict at work	-	−0.10	−0.02	−0.01	−0.01	0.01	0.03	(−0.46, 0.51)	0.24	0.01	0.843
Organisational constraints	-	-	−0.22*	−0.22*	−0.19	−0.18	−0.15	(−0.32, 0.01)	0.08	−0.20	0.075
Age	-	-	-	0.08	0.09	0.09	0.11	(−0.05, 0.26)	0.08	0.12	0.195
Death and dying-related stress	-	-	-	-	−0.13	−0.10	−0.10	(−0.41, 0.21)	0.16	−0.07	0.491
HIV and AIDS stigma by association	-	-	-	-	-	−0.10	−0.29	(−0.56, −0.01)	0.14	−0.21*	0.046
Job status	-	-	-	-	-	-	−1.94	(−3.12, −0.76)	0.59	−0.32**	0.001

*Source:* Authors’ own work

*N* = 110.

*β*, beta; SE *β*, standard error of beta; CI, confidence interval; *P*, significance.

* , *p* < 0.05;

** , *p* < 0.01

#### Emotional exhaustion

The regression model explained nearly 40% of the variance in emotional exhaustion (*R*² = 0.39, *F*(7, 102) = 9.28, *p* = 0.00), a medium effect size in the social sciences. Workload accounted for 24% of the variance (Δ*F*(1, 108) = 34.45, *p* = 0.001). Job status and interpersonal conflict at work were also significant predictors (*p* = 0.009) of emotional exhaustion, explaining 4% and 7% of the variance respectively. Organisational constraints was the final significant predictor, accounting for 3% of the variance in emotional exhaustion. Three of the four predictor variables contributed significantly to the variance in emotional exhaustion. These were interpersonal conflict at work (*β* = 0.20, *p* = 0.024), job status (*β* = 0.23, *p* = 0.009) and workload (*β* = 0.30, *p* = 0.001).

#### Depersonalisation

The combination of predictor variables explained one-third of the variance in depersonalisation (*R*² = 0.33, *F*(7, 102) = 7.22, *p* = 0.001). The combination of interpersonal conflict and workload explained 14% and 7% in the variance respectively. However, only interpersonal conflict at work (*β* = 0.27, *p* = 0.004) was a significant predictor of depersonalisation. Additionally, organisational constraints (*β* = 0.21, *p* = 0.034), HIV/AIDS stigma by association (*β* = 0.26, *p* = 0.006) and job status (*β* = 0.21, *p* = 0.021) significantly predicted the variance in depersonalisation.

#### Personal accomplishment

The regression model significantly explained nearly one-fifth of the variance in personal accomplishment (*R*² = 0.18, *F*(7, 102) = 3.12, *p* = 0.001). Job status and organisational constraints accounted for 9% and 4% of the variance. HIV and AIDS stigma by association (*β* = −0.21, *p* = 0.046) and job status (*β* = −0.32, *p* = 0.001) significantly predicted personal accomplishment.

## Discussion

Interpersonal conflict at work was significantly positively associated with two factors of burnout, namely depersonalisation and emotional exhaustion. A relationship between burnout and interpersonal conflict at work has also been found in studies (Guidroz et al. 2012). One consequence of burnout among nurses is the potential for acrimonious relationships with patients, which can have far-reaching implications in terms of the provision of care. For example, South African patients reported that acrimonious relationships with nurses led some to cease their clinic visits, eventually leading to their abandoning clinic care and consequent poor health outcomes (Coetzee et al. 2011). To this extent, burnout among nurses is a factor in determining the experience of patients receiving healthcare services (Garman, Corrigan & Morris 2002). When patients drop out of care as a result of negative experiences with nurses, this may impact their adherence to care, including antiretroviral medication, resulting in opportunistic illnesses and ultimately death. Thus, burnout among nurses may have devastating consequences for patient well-being.

Yet, the relationship between burnout and patient-nurse interaction is likely to be bidirectional. For example, one study found that among nurses and police personnel, emotional job demands, including difficult relationships with patients in the case of nurses, explained a significant proportion of the variance in burnout (i.e. exhaustion and cynicism or disengagement). This relationship was mediated by emotional dissonance (Bakker & Heuven 2006). Emotionally demanding interactions with patients may result in emotional dissonance which in turn may lead to job burnout and impaired performance.

Burnout also has implications for nurses’ work attendance, which is closely related to the quality of patient care (Peterson et al. 2008).

Public hospitals in low- and middle-income countries such as South Africa are characterised by a high volume of patients and a disproportionately low number of nurses to serve them. It is likely that the elevated levels of the various dimensions of burnout are related to the experience of being overwhelmed by the patient load.

### Burnout as a function of workload

Interpersonal conflicts at work, organisational constraints, workload, job status and HIV and AIDS stigma by association were identified as significant predictors of burnout among our sample. These predictors accounted for 32% of the variance in depersonalisation, 38% of the variance in emotional exhaustion and 12% of the variance in personal accomplishment. All predictor variables, with the exception of age, were significantly associated with levels of burnout.

Nurses’ workload has been directly associated with burnout in other studies (Laschinger et al. 2012). This finding is similar to ours in that workload was a significant predictor of both the depersonalisation and emotional exhaustion aspects of burnout. Similar to our results, Garrosa et al. (2008) found that among workload, personality characteristics, sociodemographic characteristics and job stressors, workload was the most significant predictor of emotional exhaustion. In addition, workload was associated with high levels of burnout in nurses in South Africa (Coetzee et al. 2013; Görgens-Ekermans & Brand 2012).

Job status was significantly negatively correlated with the personal accomplishment dimension of burnout and significantly positively correlated with the emotional exhaustion dimension of burnout. Job status accounted for 4%, 7% and 9% of the variance in depersonalisation, emotional exhaustion and personal accomplishment, respectively. A higher job status was associated with higher levels of burnout. These findings are similar to those of Garrosa et al. (2008), and Van der Colff and Rothmann (2012).

Organisational constraints was significantly negatively associated with the personal accomplishment aspect of burnout and significantly positively associated with the depersonalisation and emotional exhaustion aspects of burnout. Other studies also found significant relationships between levels of burnout in South African nurses and organisational constraints (Coetzee et al. 2013; Engelbrecht et al. 2008; Van der Colff & Rothmann 2009).

The occupational stressor HIV and AIDS stigma by association was negatively associated with the personal accomplishment aspect of burnout and positively associated with the depersonalisation aspect of burnout. Existing literature shows that HIV and AIDS stigma by association experienced by nurses is a significant occupational stress factor (Chirwa et al. 2009; Haber et al. 2011). Death and dying-related stress were significantly negatively associated with the personal accomplishment aspect of burnout and significantly positively associated with the depersonalisation and emotional exhaustion aspects of burnout. Yet, we found only one study supporting a significant relationship between levels of burnout among nurses and death and dying-related stress (Garrosa et al. 2008). Conflictual interaction and workload were the other predictors of depersonalisation and emotional exhaustion, whereas role ambiguity and conflictual interaction were the other predictors of personal accomplishment.

Because of the limited sample size of 109, the different sources of interpersonal conflict could not be investigated separately, as the sample size could not accommodate three additional predictor variables. A second limitation was that participants were selected from one public hospital in the Western Cape and findings can therefore not be generalised to the entire population of South African nurses. A third limitation was that the participants may have experienced different levels of occupational stress factors as they were recruited from different parts of the hospital.

## Conclusion

We found that burnout was elevated among the sample. We recommend developing and testing interventions aimed at reducing burnout among nurses. For example, reducing workload may result in ameliorating burnout, although the ratio of nurses to patients is a direct result of budgets available for hospital staff. It is possible that interpersonal conflict at work may decline with lower levels of burnout, although such a relationship requires testing. Our data provide no information on the directional relationship between burnout and interpersonal conflict and thus it is not known whether burnout causes conflict, whether it occurs as a consequence of conflict or whether causality is bidirectional. Opportunities for promotion and for acknowledgement of professional accomplishments may also serve to reduce the likelihood of burnout, although these again would be possible only in the context of available financial and other resources. Surprisingly, death and dying-related stress were not found to be associated with levels of burnout. Considering our findings that interpersonal conflict contributed to burnout among the sample, the sources of such interpersonal conflict require further study so as to reduce the likelihood of this occurring.

## Recommendations

Several recommendations may be made from our results. Firstly, given the robust association between burnout and interpersonal conflict at work, it is likely that nurses may benefit from an employee wellness programme that includes activities such as dispute resolution and the creation of a positive and supportive work environment. Secondly, consideration needs to be given to ways in which workload may be reduced. For example, ART adherence clubs for highly adherent patients, which have been implemented in some clinics in the Western Cape, have resulted in a more streamlined process where patients are seen by a nurse and pharmacist within 1 h (Dudhia & Kagee 2014). Thirdly, by all accounts, nurses require additional support to manage emotionally demanding patients. Given elevated rates of common mental disorders among persons receiving ART, it is likely that many patients may benefit from additional psychological support. Thus, professional counsellors may assist by attending to patients who require emotional and psychological help. Finally, the issue of dealing with death and dying may also be addressed by employee wellness programmes.
